# Mouse DRG Cell Line with Properties of Nociceptors

**DOI:** 10.1371/journal.pone.0128670

**Published:** 2015-06-08

**Authors:** Ciara Doran, Jonathan Chetrit, Matthew C. Holley, David Grundy, Mohammed A. Nassar

**Affiliations:** Department of Biomedical Science, University of Sheffield, United Kingdom; University of Texas at Dallas, UNITED STATES

## Abstract

*In vitro* cell lines from DRG neurons aid drug discovery because they can be used for early stage, high-throughput screens for drugs targeting pain pathways, with minimal dependence on animals. We have established a conditionally immortal DRG cell line from the Immortomouse. Using immunocytochemistry, RT-PCR and calcium microfluorimetry, we demonstrate that the cell line MED17.11 expresses markers of cells committed to the sensory neuron lineage. Within a few hours under differentiating conditions, MED17.11 cells extend processes and following seven days of differentiation, express markers of more mature DRG neurons, such as NaV1.7 and Piezo2. However, at least at this time-point, the nociceptive marker NaV1.8 is not expressed, but the cells respond to compounds known to excite nociceptors, including the TRPV1 agonist capsaicin, the purinergic receptor agonist ATP and the voltage gated sodium channel agonist, veratridine. Robust calcium transients are observed in the presence of the inflammatory mediators bradykinin, histamine and norepinephrine. MED17.11 cells have the potential to replace or reduce the use of primary DRG culture in sensory, pain and developmental research by providing a simple model to study acute nociception, neurite outgrowth and the developmental specification of DRG neurons.

## Introduction

The cell bodies of sensory neurons of the peripheral nervous system reside in the cranial and dorsal root ganglia (DRG). Sensory neuron function is altered in response to the endogenous release of inflammatory mediators in myriad pathological conditions [1]. DRG neurons in primary culture have been used to study the molecular mechanisms of acute nociception and peripheral sensitisation as well as to screen for drugs targeting these pathways. The drawbacks of primary culture include limited material requiring large numbers of animals to be sacrificed, labour intensive isolation procedures, poor transfection efficiencies, heterogeneity of cytochemical phenotypes and the presence of non-neuronal cells that confound “omic” studies. Several DRG cell lines have been generated, including the rat DRG/mouse neuroblastoma hybrid cell lines [2,3] and a rat embryonic DRG cell line [4]. But given the large number of DRG neuron subpopulations, more cell lines are required to represent the diversity of phenotypes. Moreover since the development of transgenic and gene knockout technology, there has been an increased reliance on murine models to study the mechanisms of acute and pathological pain and peripheral nervous system development. To date, no murine DRG cell lines exist to complement such studies. Recently, cells with nociceptive properties were derived from human pluripotent stem cells (hPSCs) using combined small molecule inhibition [5,6]. However, the reported protocols involve painstaking maintenance and manipulation of stem cells and require up to seven weeks for the emergence of some nociceptive markers. For these reasons, we set out to create mouse DRG cell lines with nociceptive properties and to develop an efficient differentiation protocol. We used the Immortomouse [7] to clone immortalised sensory neuron progenitors. The Immortomouse expresses a thermolabile simian virus 40 large T antigen tsA58. The transgene is under the control of the Major Histocompatibility Complex (MHC) H-2K^b^ Class I promoter, which is basally active in many tissues and can be further induced by interferon. At 33°C, the large T antigen tsA58 is stable, but at 39°C the protein is rendered non-functional. The Immortomouse has been used to create many conditional cell lines from mitotic cells. To increase the likelihood of isolating neurons of the nociceptive lineage, we isolated several lines from embryonic day E12.5 DRG, a developmental stage when proprioceptive and low threshold mechanoreceptive-lineage neurons have terminally differentiated but nociceptive lineage neurons are still dividing. Here we present the derivation and characterisation of the Mouse Embryonic DRG (MED) cell line, MED17.11. These cells express markers of committed sensory neuron progenitors. However, when cultured in our differentiation medium, they express markers of maturing DRG neurons including numerous ion channels. We also observed functional responses to noxious compounds and inflammatory mediators. Therefore the MED17.11 cells may provide a simple model to study both acute nociception, developmental specification of DRG neurons and potentially the mechanisms of peripheral sensitisation.

## Materials and Methods

### Animals and DRG culture

A small Immortomouse mouse colony was maintained by the university of Sheffield Biological service unit. Breeding and maintenance of the mouse colony was carried out under Home Office Project License PPL 40/3430. Mice of all ages were sacrificed using a humane method as listed in Schedule 1 of the Animal (Scientific procedure) Act 1986. E12.5 embryos from the H2kbtsA58 Immortomouse were killed by immersion in ice-cold PBS followed by decapitation. DRG from all vertebral levels were collected into PBS on ice and tail tips were harvested for genotyping for the presence of the temperature-sensitive SV40 large T antigen (TSA58-sense 5’-TGCCAGGTGGGTTAAAGGAGCATGA-3’ and TSA58-antisense 5’-AGCCAAGCAACTCCAGCCATCCA-3’). The DRG were digested for 40 minutes in a mixture of 0.6 mg/ml collagenase type IX (Sigma) and 1 mg/ml Dispase II (Gibco) in enzyme incubation solution [8] at 37°C in 5% CO_2_ and 95% air. Following trituration, the DRG were resuspended in medium permissive for T antigen expression. This comprised a basal medium of DMEM/F12 with stable glutamine, penicillin and streptomycin and 10% FBS (all PAA), supplemented with interferon gamma (100 units/ml during initial establishment of DRG cell lines and reduced later to 50 units/ml), chick embryonic extract at 0.5% (Sera Labs) was added to augment the proliferation rate. The cells were cultured at 33°C for a few passages before cloning.

### Establishment of DRG Cell Lines

Individual clones were isolated by cloning rings. We observed that cloning by limited dilution in multi-well plates lead to differentiation and death of cells. Primary clones were subjected to an initial screen to select for βIII-tubulin (Tuj1) immunoreactivity. These primary clones were subcloned until homogeneity of shape and Tuj1 expression was observed. The cell lines were routinely cultured in permissive medium (proliferating conditions) and passaged weekly. We have selected for clones with robust growth over more than 100 passages.

### Differentiation in Non-Permissive Conditions

Before all experiments, the cells were plated onto polyornithine-coated glass coverslips or tissue culture-treated plastic and transferred to non-permissive medium for T-antigen expression overnight (medium as above, but without interferon gamma and chick embryonic extract). The large T antigen protein is rendered non-functional at 39° C [7]. However, this temperature is close to the thermal activation threshold of the heat and capsaicin receptor, TRPV1 (~42°C) which is expressed early in developing DRG [9], so to avoid activation of the ion channel, the cells were maintained at 37°C.

### Immunocytochemistry

Cells were washed in ice-cold phosphate buffered saline (PBS) and fixed in 4% paraformaldehyde for 10 minutes. Paraformaldehyde autofluorescence was quenched by incubation with 50 mM ammonium chloride for 20 minutes. The cells were permeabilised for 15 minutes in PBS with 0.1% TritonX-100, washed and placed in blocking solution for one hour at room temperature prior to overnight incubation with primary antibodies at 4°C. Following, three washing steps in PBS, the cells were incubated with DAPI (1:5000) and Alexa Fluor anti-mouse or anti-rabbit secondary antibodies for two hours at room temperature (1:2000 each, Life Technologies). After another three washing steps, coverslips were mounted on slides using Prolong Gold (Life Technologies). Antibodies were verified in adult primary DRG in culture. We used monoclonal antibodies for Tuj1 (1:1000, R &D), CNPase (1:250, Abcam) and GFAP (1:250, Sigma). The polyclonal antibodies used were Isl1 (1:250, Abcam), FOX3 (NeuN, 1:250, Millipore), SOX10 (1:250, Abcam), Advillin (1:500, Abcam), TrkA, TrkB and TrkC (1:500, Alomone), Nav1.3 (1:500, Alomone), and SV40 (1:250, Santa Cruz).

### RT-PCR

RNA isolation was performed using TRI-reagent (Sigma) and Direct-zol RNA mini-prep (Zymo Research) according to the manufacturers’ instructions. Primers were designed to flank exon junctions and across two exons using NCBI Primer BLAST (Table 1). Reverse transcription (2000 ng input RNA) was performed using High Capacity cDNA Reverse Transcription Kit (Applied Biosystems) with random hexamers. cDNA was amplified by PCR using Go Taq polymerase with 5x Green buffer (Promega). The synthesised cDNA (50 ng of calculated from original RNA) was used as a template for RT-PCR, using primers summarised in Table 1. Following initial denaturation at 95°C for 10 minutes; the cDNA was amplified for 35 cycles using the following parameters: 95°C for 45 seconds; 57°C for 30 seconds, 72°C for 30 s. A final extension was performed at 72°C for 10 minutes. Primers were validated with cDNA synthesised from adult and E13.5 DRG mRNA.

**Table 1:** Primer Sequences 5'-3'.

Target	Sense Primer	Antisense Primer	Accession Number
SOX10	TGCTGCTATTCAGGCTCACTACAA	CAGGTATTGGTCCAGCTCAGTCAC	NM_011437.1
P75	GGGCACATACTCAGATGAAGCCAA	CAGCAGCCAAGATGGAGCAATAGA	NM_033217.3
FOXS1	GGCATCTACCGCTACATCATGGG	ACAGTCTGCCAGTTGTGGTCTTG	NM_010226.2
Advillin	GGGTCAGTTCCAGGAAGACAGC	GAAGTAGCCACGGAAGGTGTCG	NM_009635
Brn3A	CCACTTACTGAGGAGGGTGTGAGA	TGTGACTCAACATTTATCCCCGGT	NM_011143.4
cRET	AGGAAATGTACCGTCTGATGCTGC	TACAGAGAGTTGGGATGGTGCAGA	NM_001080780.1
TrkA	GGGAGTTGAGAAGCCTAACCATCG	CAGAGTCATTGGGCATCTGGATCT	NM_001033124.1
TrkB	CAATGAGAGCAGCAAGAACATGCC	GATAGTTGGCGCAAAATGCACAGT	NM_001025074.2
TrkC	TTACTACAGGGTGGGAGGACACAC	AAGAATGTCCAGGTAGATCGGGGT	NM_008746.5
Runx1	GGTAGCGAGATTCAACGACCTCAG	ATCCTGCATCTGACTTTGAGGCTG	NM_001111021.2
Runx3	AGCCAACCAAGTGGGTCTGAAC	AGCACGGAGCAGAGGAAGTTG	NM_019732.2
TRPV1	ACATCTGGAAGCTGCAGCGAG	TTGCGTCCCTCAGAAGGGGA	NM_001001445.1
TRPV2	ACTGGGCCAGCTGTGGTACT	GCCTCCCGGCTCAAGCTTAC	NM_011706.2
TRPA1	CCTGCTTCACAGAGCCTCGTTATT	GCCTACAGGCATAATGGAGAGGTG	NM_177781.4
Piezo1	TCATCATCTCTAAGAATATGCTGTCGCTC	CAATCTGGCGATGGAAGTTGATG	NM_001037298.1
Piezo2	CTCTGTGTCCTGCTGGCAATCTTCA	CCTCTCTGCCGTGTTCTGATTGGAG	NM_001039485.4
CGRP	CGCTCACCAGGAAGGCATCAT	GTTGTCCTTCACCACACCTCCTG	NM_001033954.3
Nav1.9	ATCCCAAGGCCCCTGAACAAA	GTGTGGGCGGGAAGACGTTG	NM_011887.3
	ATTCCAGAGGGAAAGATGAGCAGC	TCAAAGACCTGGCTTGTGACCAAA	
Nav1.8	AACAATACTGGCCACTTCTTCTGGG	CATGAAGATGTCCTGGCCCCTTAT	NM_009134.3
Nav1.7	AGCAGGAAGAAGCCGAGGTAGTAT	AATGCTGAGTGGTGACTGGTTGG	NM_018852.2
	TTCCGAGGCCAGGGAACAAAT	GCGAATGACTCGGAACAGGGT	

### Calcium Imaging

Cells were loaded with 2 μM Fura2-AM (Molecular Probes) for 30 minutes and allowed to recover in standard extracellular solution for a further 30 minutes before experiments. All recordings were performed at room temperature. The cells were superfused with standard extracellular solution for at least 5 minutes before beginning recordings. Ratiometric measurements of intracellular calcium [Ca^2+^]i were made using a Cairn Dual OptoLED (excitation wavelengths: 350 and 380 nm) with a Hamamatsu C4742-95 camera and *Simple PCI 6* software (Hamamatsu). Background subtraction and ratio calculations (350/380 nm) were performed within the software. Standard extracellular solution contained the following (mM): NaCl (140); KCl (4); CaCl_2_ (2); HEPES (10); NaOH (4.54) and glucose (5). For high potassium extracellular solution, the concentration of KCl was increased to 40 mM and NaCl was reduced to 104 mM. Both solutions were adjusted to pH 7.4 at room temperature using NaOH. We tested three fields of cells for each compound. To avoid desensitisation mechanisms, only one stimulus was applied per coverslip, unless the cells failed to respond, in which case another drug was tested on the same coverslip.

### Compounds

All drugs were applied in standard extracellular solution from the following stock solutions: capsaicin (10 mM in ethanol), veratridine (50mM in ethanol), WS-12 (10 mM in DMSO), ATP (2mM in H_2_O), bradykinin (0.5 mM in H_2_O), serotonin (5mM in H_2_O), histamine (1mM in H_2_0), norepinephrine (0.5 mM in HCl). Solvents were added to the control standard extracellular solution at the same final concentration as the drug solution.

### Data Processing and Statistical Analysis

Statistical analysis was performed using *GraphPad Prism 6* software and *SigmaPlot version 12*.*0*. For the calcium imaging data, cells that responded with a rise in fluorescence ≥ ΔF/F_0_ = 0.1 were considered to be responders.

Cell surface area measurements were performed in *Image J*. A free-hand region of interest was drawn around the perimeter of the soma. The cell diameters were estimated from the area, which was assumed to be equal to πr^2^.

### Transient Transfection

Transient transfection with pmaxGreenFP was performed using Lipofectamine LTX with the Plus reagent. Cells were seeded at density of 12500/cm^2^ for transfection the following day. For each cm^2^ the following was added in antibiotic free medium: 0.25 μg of DNA, 0.25 μl of plus reagent and 0.75 μl lipofectamine LTX were used. Transfection was allowed to proceed overnight.

## Results

### Initial screen of immortalised Tuj1 positive clones

We selected dividing cells for cloning from E12.5 cultures on the basis of immunoreactivity for the neuron-specific marker Tuj1 and then selected 28 clones based on robustness of proliferation and uniformity of Tuj1 expression. Using RT-PCR and immunocytochemistry we screened these 28 clones for markers of neural crest cells, glia and post-mitotic sensory neurons, both in proliferating conditions and following differentiating conditions (data not shown). MED17.11 cells were identified as having an advanced sensory neuron-like profile coupled with a strong proliferation capacity. This clone was chosen for further detailed characterisation.

### Proliferating MED17.11 cells express neuronal markers and can be transfected

**Fig 1:**
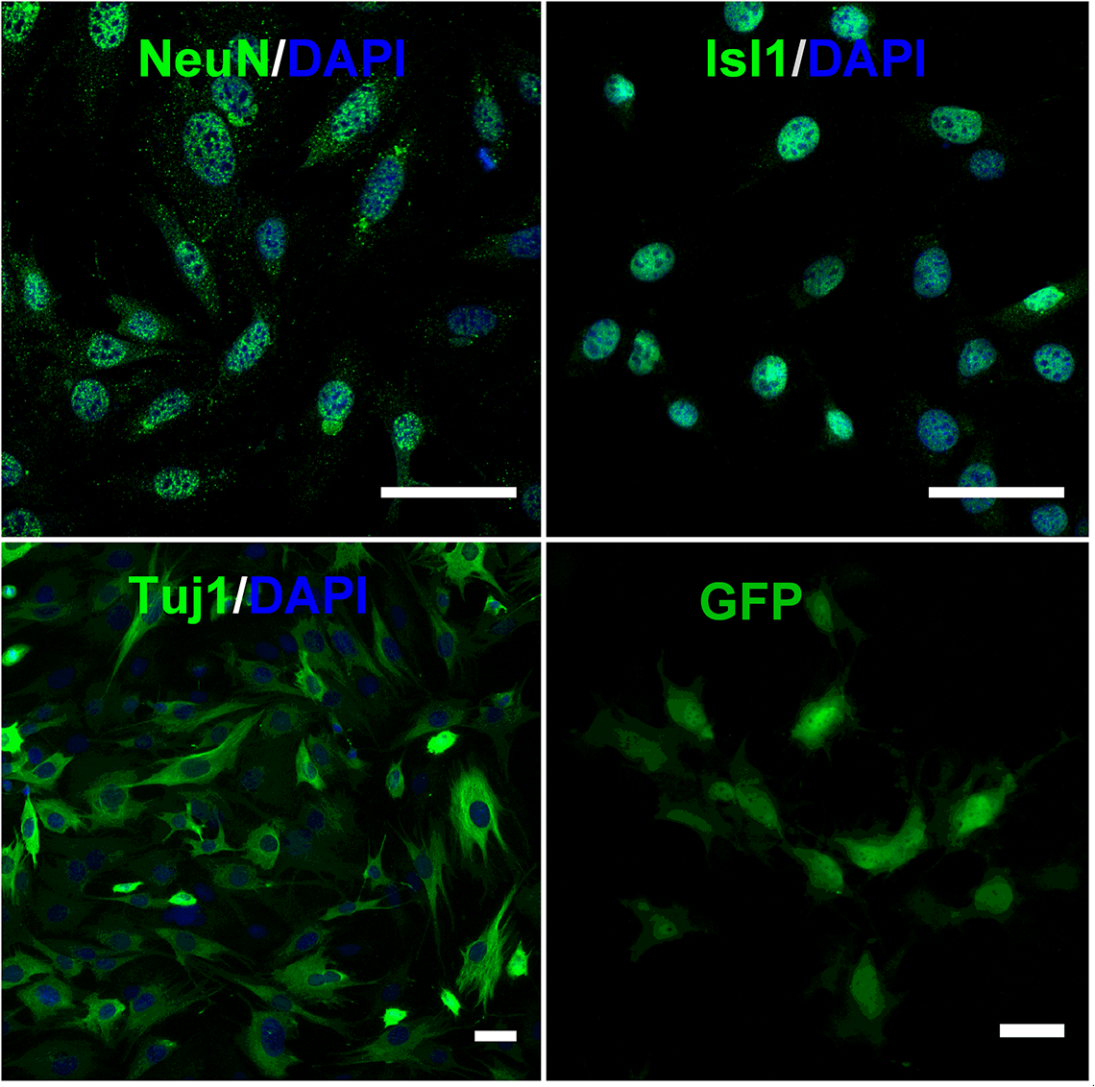
MED17.11 express the early neuronal markers FOX3 (NeuN), Isl1 and Tuj1, and can be transfected with GFP. Immunolabelling of MED17.11 cells cultured in permissive conditions for large T antigen expression. GFP transgene expression in MED17.11. Scale bar is 100 μm.

When maintained in proliferating conditions, the cells adopted a flattened morphology (Fig 1) and were immunopositive for the generic neuronal markers Tuj1 and FOX3 (NeuN, Fig 1). MED17.11 cells were also immunopositive for the LIM homeodomain transcription factor Isl1 (Fig 1), which plays a role in sensory neuron survival and maturation, in particular for nociceptors [10]. Importantly, MED17.11 was negative for the glial markers GFAP, SOX10 and CNPase (data not shown).

Screens involving gene over-expression or knockdowns in primary DRG neurons in culture are difficult, owing to the challenge of obtaining high quantities of purified neurons, poor transfection efficiencies, particularly with standard lipid based transfection reagents which can cause a loss of cell viability. As proof of principle we have demonstrated that MED17.11 cells can be transfected using a lipofection-based reagent (Fig 1, GFP).

### Differentiated cells rapidly adopt a neuronal morphology and express the DRG neuron marker Advillin

**Fig 2:**
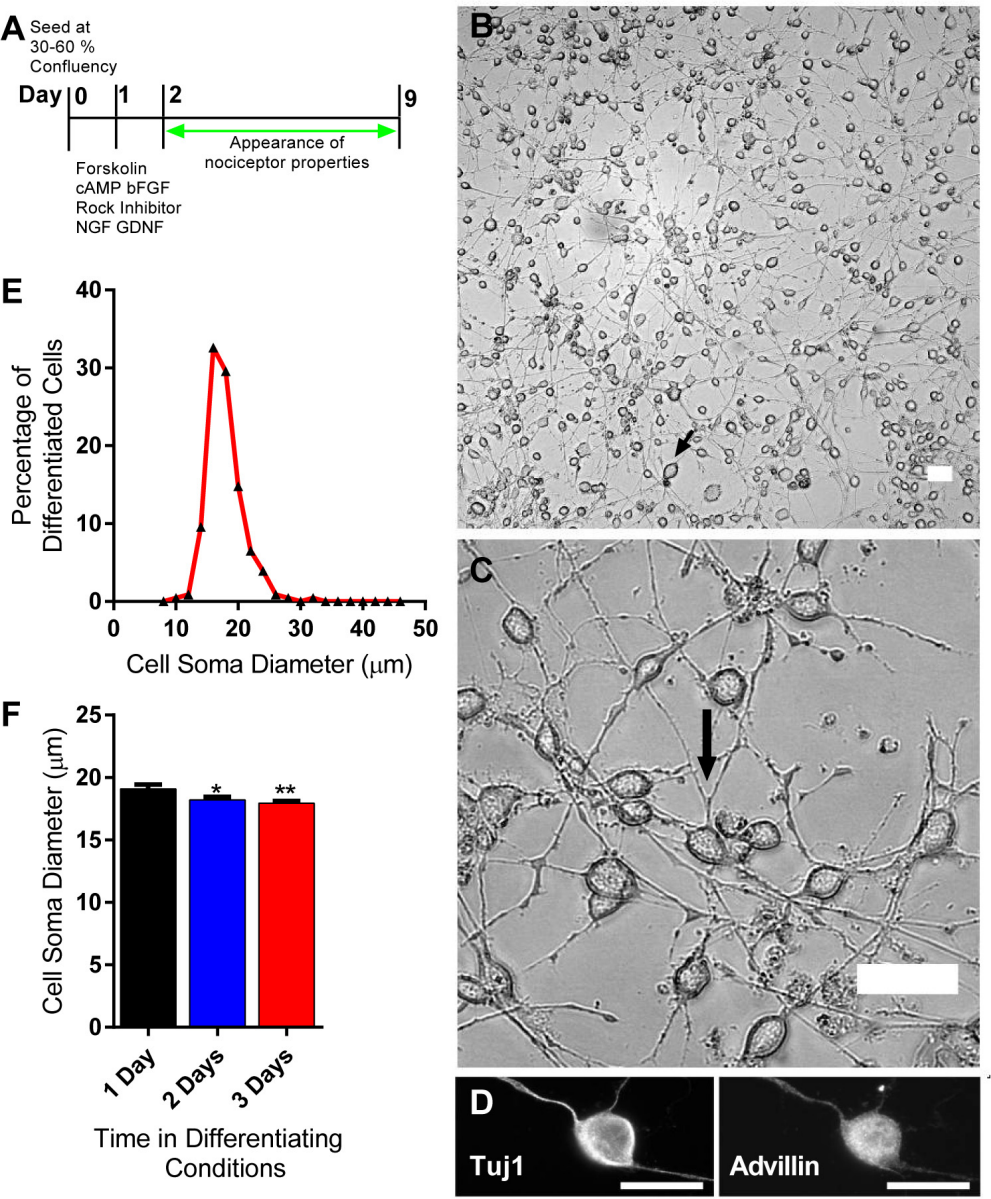
Morphological differentiation of MED17.11. A, Protocol for differentiation of MED17.11 cells. Bright-field images (B and C) of MED17.11, 3 days after the addition of differentiation medium. B, Low magnification images illustrate the high efficiency of morphological differentiation using the protocol shown in A. The small black arrow points to the rarer large diameter neurons. C, Higher power images showing the typical morphology of MED17.11 cells. The large black arrow points to an example of process branching. D, Frequency distribution of cell soma diameters (left) demonstrates the diversity of cell sizes following differentiation. E, Histogram showing change in soma diameter post differentiation (right) * = p<0.05, ** = p<0.001 (mean + S.E.M; Student’s unpaired two tailed t test). F, Immunofluorescence images of MED17.11, 3 days after the addition of differentiation medium. Both soma and processes of MED17.11 cells label with the neuronal marker, Tuj1, and the sensory neuron marker, Advillin. All scale bars are 50 μm.

The protocol for differentiation is represented in Fig 2A. Our differentiation medium contained bFGF (10 ng/ml, R&D Systems), di-butryl cAMP (0.5mM, Sigma), forskolin (25 μM, Cell Signalling Technology) [11] and rock inhibitor Y-27632 (5 μg/ml, Chemdea), which promotes neurite outgrowth in embryonic DRG [12] and induces neural crest cell differentiation [13]. Growth factors NGF (100 ng/ml) and GDNF (20 ng/ml, both R&D) were also added to the differentiating medium, the former being required for nociceptor survival and both playing a role in phenotype specification. Within hours of switching to the differentiation medium, the majority of cells displayed a bipolar morphology typical of immature DRG neurons [14]. Fig 2B and 2C are bright-field images after three days in differentiating medium (day 5 of the differentiation protocol, Fig 2A). The majority of cells had a phase-bright rounded soma with two processes which extended in length and branched extensively in culture. We found that morphological differentiation was strongly dependent on cell density and was optimal when the cells were plated at 5000-15000/cm^2^. At higher densities a smaller percentage of cells showed visible differentiation and at lower densities the survival rates were poor.

MED17.11 cells displayed some heterogeneity in terms of cell size. Fig 2D shows the frequency distribution of soma diameters measured on day 5 of the differentiation protocol. Cell body diameters ranged from 8 to 32 μm, but 77.8% of MED17.11 cells were between 14 and 20 μm, with a mean diameter of 18.2 ± 0.2 μm (n>200 cells). The cell diameters showed a small but significant decrease over days post-differentiation (Fig 2E). This is attributable to the loss of a small population of larger diameter cells over this period. The soma size is equivalent to small diameter adult neurons, but larger than reported in mouse at E15.5 [15] where TrkA-positive (nociceptive) and RET positive (early RET expressing, mechanoreceptive, population) neurons had a mean diameter of 10.1 and 15.5 μm respectively (reported as surface area = 80.6 ± 1.8 μm^2^ and 187.8 ± 7.6 μm^2^; diameter calculated assuming area = πr^2^). MED17.11 cells were uniformly immunoreactive for the sensory neuron marker Advillin (Fig 2F) which is selectively expressed in rat DRG and superior cervical ganglia [16] and has even more restrictive DRG expression in mouse [17].

### MED17.11 cells are committed to the sensory neuron lineage and mature when differentiated

**Fig 3:**
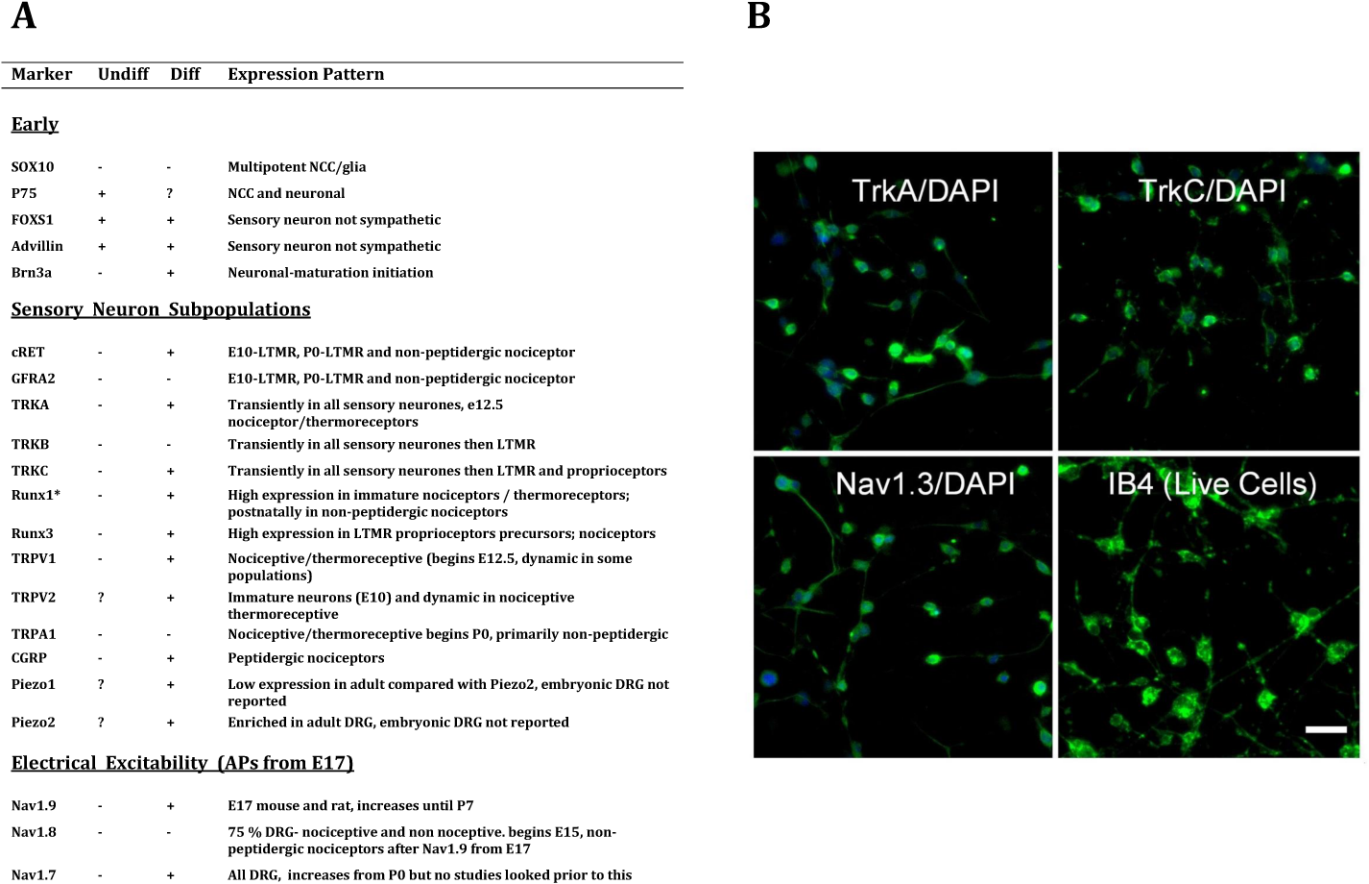
mRNA and Immunocytochemical profiles of MED17.11. Left, mRNA analysis of proliferating and differentiated MED17.11 cells: “Undiff” refers to cells grown in proliferating conditions (see methods). “Diff” refers to cells grown in differentiating conditions for 7 days (day 9 of differentiation protocol in Fig 2, also see methods). A “+” indicates that the cell line was positive and a “-” indicates that the cell was negative for the corresponding marker. A”?” indicates that the marker was not tested in the given condition A. The “*” beside Runx1 indicates that this nociceptor marker was also tested after one day in differentiation conditions and was positive. LTM = low threshold mechanoreceptor, VGSC = voltage gated sodium channel. Right, immunocytochemical analysis of MED17.11 cells differentiated for 7 days. The cells showed immunoreactivity for the modality specific markers TrkA and TrkC (top) and the embryonic VGSC, Nav1.3 (bottom left). Live MED17.11 cells also strongly labelled with IB4 (bottom right). Scale bar is 50 μm.

To understand whether morphological differentiation by our medium was coupled to cytochemical maturation, we screened the cells for markers of sensory neurons and neural crest cells using RT-PCR in proliferating and differentiating conditions (7 days, which is day 9 of the differentiation protocol, Fig 2A). We included some markers that are expressed in modality-specific subpopulations of DRG and whose expression pattern has been described in embryonic DRG. However, the primary focus was on markers of nociceptive neurons. The mRNA phenotype is summarised in Fig 3 and suggests that the undifferentiated cells are already committed to the sensory neuron lineage, but mature when cultured in our differentiation medium as described below.

In agreement with immunocytochemical analysis, in proliferating conditions, we did not detect mRNA for SOX10, a marker of multipotent neural crest cells whose expression is subsequently maintained only in glial cells [18]. Importantly, we detected expression of FOXS1, which is restricted to sensory neuron committed cells and is not expressed in the sympathetic chain in E12.5 mice [15]. In agreement with immunocytochemical analysis, we also detected mRNA for Advillin in both proliferating and differentiated cells. Taken together this suggests that the phenotype of MED17.11 cells correlates well with committed sensory neuron progenitors.

Within peripheral sensory neurons, RUNX1 is selectively expressed in developing nociceptive lineage neurons and maintained in the non-peptidergic nociceptor population postnatally [19] [20]. After just one day in differentiating conditions MED17.11 cells expressed this transcription factor (Fig 3). TrkA is initially found in all nociceptors/thermoreceptors (but not large diameter neurons), but postnatally, it is down-regulated in half of the population. These neurons start to express the GDNF receptor, cRET, do not synthesise peptides such as CGRP and substance P and label with the plant isolectin, IB4 [15]. Differentiated MED17.11 expressed mRNA for cRET; interestingly, we also detected mRNA for CGRP, a marker of peptidergic nociceptors. This suggests that MED cells are capable of differentiating into different subpopulations of DRG neurons (note that the differentiation medium contains both NGF and GDNF). We also detected mRNA for TRPV2, which is expressed in post-mitotic DRG neurons and developing motor neurons from E11, but not in other spinal cord neurons [20]. Of note, we also detected mRNA for the mechanosensor proteins, Piezo1 and Piezo2. Both are expressed in DRG, with Piezo 2 being highly enriched in adult DRG [21] but their expression profiles have not been reported in embryonic DRG.

To determine whether the cells expressed markers of proprioceptor/mechanoreceptor lineage neurons, we looked for expression of receptor tyrosine kinases TrkB and TrkC as well as the runt domain transcription factor Runx3. When differentiated MED17.11 expressed TrkC and Runx3 (Fig 3).

Voltage gated sodium channels (VGSCs) confer electrical excitability to adult sensory neurons with NaV1.7, NaV1.8, and NaV1.9 being important molecular substrates underlying the excitability of nociceptors in DRG neurons. Following differentiation, we could not detect mRNA for Nav1.8 at this time-point (7 days in differentiating conditions), however, the cells expressed mRNA for VGSC NaV1.7 whose expression in sensory neurons is required for both acute and inflammatory pain [22,23] and the TTX resistant NaV1.9, which is enriched in non-peptidergic nociceptors [24,25].

To determine whether mRNA expression analysis reflected actual protein translation and to confirm the reproducibility of our protocol, we screened cells in differentiating conditions for 7 days using antibodies against trkA and trkC (Fig 3B). In agreement with mRNA analysis, both cells were immunopositive for the two receptor tyrosine kinases. While all cells were immunopositive, the level of intensity was heterogeneous, suggesting that each receptor was enriched in certain cells. Live MED17.11 cells labelled strongly with isolectin B4 (IB4, Fig 3) which, as stated above, predominantly labels small diameter, non-peptidergic neurons.

Although mRNA and immunocytochemical evidence suggests that MED17.11 cells mature in our differentiation medium, it is unclear whether they still retain properties of embryonic neurons. While the VGSC NaV1.3 is not expressed in healthy adult DRG neurons, it is highly expressed in the developing DRG [26], making it a useful indicator for the degree of neuronal maturation. Indeed, all MED17.11 cells were immunoreactive for Nav1.3 suggesting cells differentiated for 7 days are still embryonic in nature.

### MED17.11 responds to noxious compounds and the voltage-gated sodium channel agonist veratridine

**Fig 4:**
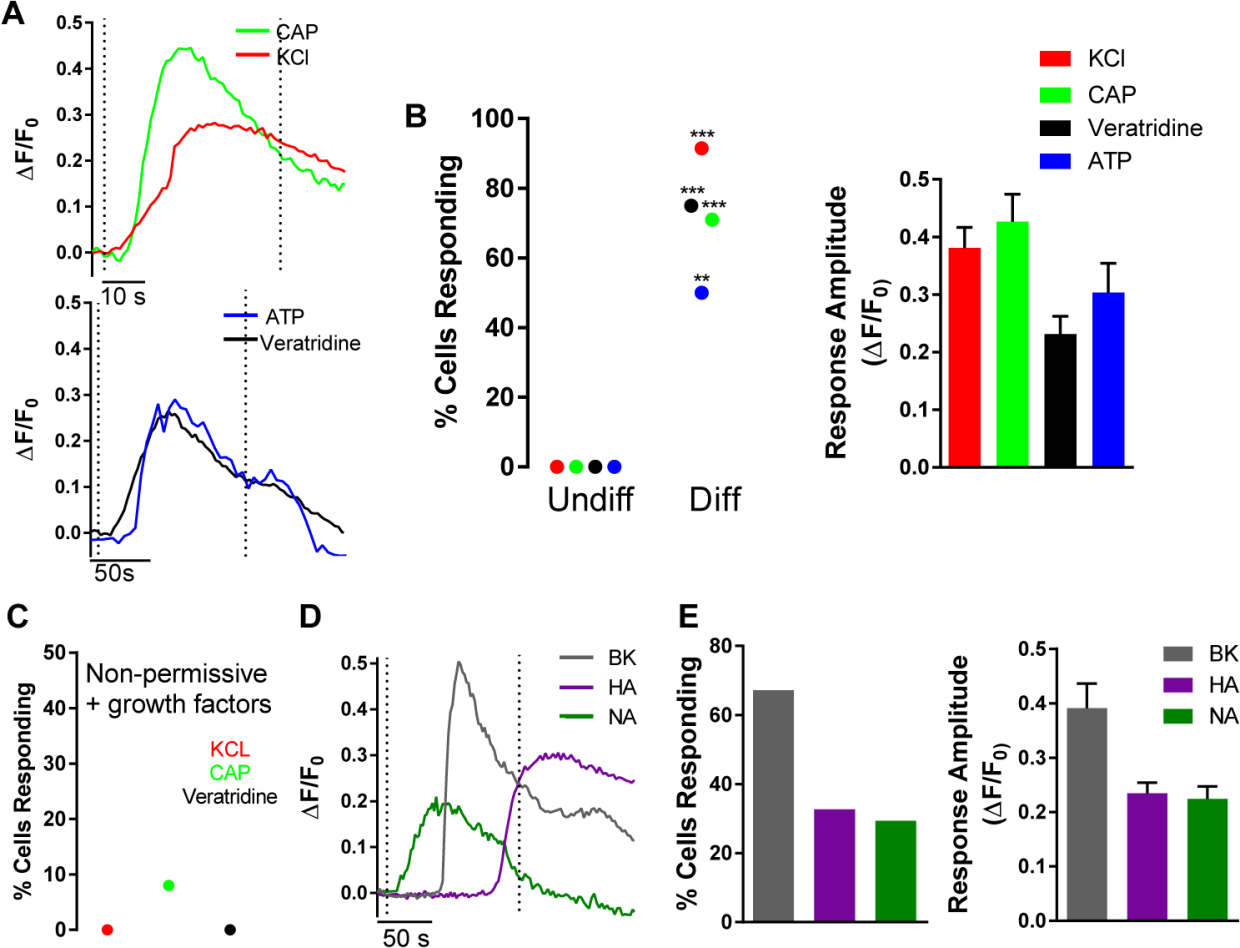
Differentiated MED17.11 responds to compounds that excite sensory neurons and inflammatory mediators. A, representative traces from fura-2 AM calcium imaging showing responses to compounds that excite DRG neurons following 3–6 days of differentiation. Dotted Lines indicate the time when the stimulus was applied. B, left, percentage of proliferative and differentiated cells responding to each compound (Fisher’s Exact two tailed test), * = p<0.05, ** = p<0.001, ***p<0.0001. Right, response amplitudes to each compound (mean + S.E.M). C, Percentage of MED cells responding when grown in non-permissive conditions with growth factors alone (NGF 100 ng/ml and GDNF 20 ng/ml) for 3 days. D, representative traces from fura-2 AM calcium imaging showing responses to inflammatory mediators following 3–6 days of differentiation. Dotted Lines indicate the time when the stimulus was applied. E, left, percentage of cells responding to each compound; right, response amplitudes to each compound (mean + S.E.M).

To investigate whether MED17.11 cells express functional receptors found specifically in nociceptors, we used Fura-2 AM calcium microfluorimetry to examine the responses of cells in proliferating and differentiating (3–6 days) conditions to compounds known to excite sensory neurons (Fig 4). Representative traces of responses are shown in Fig 4A.

In agreement with RT-PCR data, differentiated MED17.11 cells were sensitive to the TRPV1 agonist capsaicin (10 μM), a canonical nociceptor marker (Fig 4A upper). 71% of differentiated cells were responsive, compared with proliferating conditions when none responded to capsaicin. The mean capsaicin response magnitude was *Δ*F/F_0_: 0.43 ± 0.05 (Fig 4B).

KCl depolarisation of DRG neurons elicits calcium transients by activating voltage gated calcium channels, which are selectively expressed in neuronal cells of the DRG. In the developing DRG, capsaicin responses are only observed in neurons capable of being depolarised by KCl [9], therefore we expected that a large percentage of MED17.11 cells could be depolarised by KCl. Indeed, more than 90% of MED17.11 cells responded to KCl, with a mean response amplitude of *Δ*F/F_0:_ 0.38 ± 0.04 (Fig 4B). As with capsaicin, MED17.11 cells were not depolarised by KCl in proliferating conditions (Fig 4B).

DRG neurons express purinergic receptors and are activated by their endogenous ligand ATP [27]. 50% of MED17.11 cells responded to ATP (10 μM) (Fig 4B) with a mean response amplitude of *Δ*F/F_0_: 0.3 ± 0.05 (Fig 4B). Again, we did not observe responses to ATP in control cells cultured in proliferating conditions.

We next examined the cells for sensitivity to the sodium channel agonist veratridine. This alkaloid neurotoxin elicits calcium transients in primary adult DRG neurons [28] and preferentially activates TTX sensitive sodium channels by preventing the closure of the inactivation gate on open sodium channels [29]. Similar to the percentage of cells activated by capsaicin, 75% of MED17.11 cells responded to the compound with a sustained elevation in [Ca^2+^]_i_ (Fig 4A and 4B), with a mean response amplitude of *Δ*F/F_0_: 0.23 ± 0.03. Similarly to other compounds, we did not detect veratridine responses in MED17.11 cells maintained in proliferating conditions.

TrkA positive neurons give rise to innocuous thermoreceptors and noxious cold sensors. We looked for functional responses to 30 μM WS-12, a selective compound for the cold receptor TRPM8, whose expression has been reported in embryonic DRG from E16.5 onwards initially in a subpopulation of TRPV1-positive neurons [9]. We did not observe any calcium elevation in response to this drug suggesting the cells do not express functional TRPM8 (data not shown). TRPA1 is an irritant receptor that is predominantly expressed in a subpopulation of TRPV1 positive neurons [30]. Again we failed to observe responses to the TRPA1 agonist cinnamaldehyde (200 μM; data not shown).

To validate the effectiveness of our differentiation medium, we cultured MED17.11 cells for 3 days in non-permissive conditions (at 37°C and without IFNγ) with NGF and GDNF but without bFGF, cAMP, forskolin and Y-27632 (Fig 4C). We did not observe any responses to KCl or veratridine and only 8% of cells responded to capsaicin (compared to 71% in differentiation medium), thus validating the effectiveness of our differentiation medium.

### MED17.11 expresses receptors for inflammatory mediators

Inflammatory mediators released during tissue damage can directly activate nociceptors or sensitise them to subsequent stimuli leading to hyperalgesia [1]. To determine whether differentiated MED17.11 cells have the potential to be used as an *in vitro* model to study mechanisms of peripheral sensitisation, we examined their responses to several inflammatory mediators using calcium microfluorimetry. Representative traces are shown in Fig 4D. We observed responses to bradykinin (2 μM), histamine (10 μM) and norepinephrine (10 μM) but we did not observe any responses to serotonin (10 μM; data not shown).

Bradykinin elicited large calcium transients in 67.2% of cells with a response amplitude *Δ*F/F_0_: 0.39 ± 0.04; (Fig 4E). Interestingly, only 32.7% of MED17.11 cells responded to histamine (mean response magnitude *Δ*F/F_0_: 0.23 ± 0.02). Likewise, only 29.4% of cells responded to norepinephrine with a response magnitude of *Δ*F/F_0_: 0.22 ± 0.02 (Fig 4E).

## Discussion

We have derived a mouse embryonic DRG cell line, MED17.11, from the Immortomouse. The expression profile of this cell line correlates best with committed sensory neuron progenitors, and can be differentiated efficiently to have cytochemical and pharmacological properties consistent with nociceptor-lineage neurons. The cells also expressed markers of mechanoreceptor/proprioceptor lineage neurons, suggesting that MED17.11 were isolated from DRG committed cells with multimodal potential.

### Differentiated MED17.11 cells are a robust and rapid model for neurite outgrowth

The rapid and efficient induction of neurogenesis on a simple substrate of polyornithine, following differentiation (Fig 2), together with their transfectibility, could make MED17.11 a particularly useful model system for the growing field of high content analysis, which combines automated microscopy with automated analysis for chemical/genetic screens. Quantitation of neurite outgrowth is the most popular phenotypic screen for neuronal cells. Neuronal-like cell lines are used as popular model for *in vivo* neurons in such screens. However, speed and efficiency of differentiation is a specific bottleneck. Within hours of application of our differentiation medium, MED17.11 cells elaborate long processes that extend over time in culture. Moreover, unlike many widely used cell lines, such as PC-12, MED17.11 does not have a tendency to aggregate in our differentiation conditions (Fig 2B and 2C), which is a distinct advantage for high content screens.

### MED17.11 cells have multimodal potential

Using our differentiation protocol, MED17.11 cells rapidly acquire properties of peptidergic and non-peptidergic nociceptors (Fig 3). Here, we have focused on probing their nociceptive phenotype, however TrkC and Runx3 expression indicates that they may also still have the ability to differentiate into low threshold mechanoreceptors/proprioceptive lineage neurons [31]. The differentiation medium contains the neurotrophic factors NGF and GDNF, which are involved in the specification of these populations [32]. We believe that a more defined medium could direct these cells to a specific phenotype. Regardless, this multipotentiality may be useful to study the mechanisms underlying DRG subpopulation specification. Further studies in serum-free conditions and perhaps using additional growth factors such as NT3 and BDNF are needed to determine the degree to which these cells can be directed towards a specific phenotype.

### MED17.11 cells have unique phenotypes

Several other DRG cell lines exist but more are needed to better represent the full complement of phenotypes observed in the highly heterogeneous DRG. Recently, Vetter *et al* [33] characterised the endogenous calcium responses of various cell lines to compounds known to excite DRG neurons, including the rat embryonic [2] and neonatal [3] DRG/ mouse neuroblastoma fusion cell lines, F11 and ND7/23, as well as the T-antigen immortalised rat embryonic 50b11 cell line [4]. They used several compounds also tested here: bradykinin, histamine, ATP and capsaicin. Comparison with their results suggests that MED cells have unique properties. For instance, like MED17.11, F11 and 50b11 responded to ATP whereas ND7/23 cells were insensitive. Vetter *et* al did not detect responses to capsaicin in any of these cell lines, however, this contrasts previous reports for 50b11 [4]. The reasons for this are unclear, but could be related to differences in experimental parameters [33]. F11 cells lose their capsaicin sensitivity over time in culture [34], with genetic loss being a characteristic drawback of somatic cell fusion lines. Unlike MED17.11, none of these cell lines responded to histamine. F11 and ND7/23, but not 50b11 cells responded to bradykinin, with F11 showing particularly large calcium transients. MED17.11 was sensitive to bradykinin. Similarly, this is the first report of endogenous Piezo 2 expression in a DRG cell line, so it will be interesting to evaluate the mechanoreceptive properties of MED17.11 cells.

We did not detect expression of Nav1.8 using mRNA analysis. This channel is first detected at E15 in rat DRG [26], however, the onset of expression is differentially regulated within DRG subpopulations. MED17.11 strongly labelled with IB4 and in this population, Nav1.8 first appears only after Nav1.9 expression in the late embryonic period [26]. Immunoreactivity with NaV1.3 suggests that MED17.11 in our differentiation conditions for 7 days still have an embryonic phenotype. It is possible that culturing MED17.11 for longer periods could lead to the expression of Nav1.8 or that our culture conditions lacked an essential factor governing its expression. However, the mechanisms underlying induction of expression of Nav1.8 during development are unknown. We also failed to observe functional responses to the TRPA1 agonist cinnamaldehyde. Again, it is possible that the time-point chosen could be too early to detect this marker as the onset of functional TRPA1 expression occurs in the post-embryonic period (from P0 in peptidergic nociceptors and P14 in non-peptidergic nociceptors; (9)).

### A differentiation medium for rapid and efficient cytochemical differentiation

Using our differentiation medium, MED17.11 cells cultured on polyornithine rapidly acquired markers of post-mitotic sensory neurons and differentiated morphologically with high efficiency. Neural crest cell specification of DRG neurons is an active area of study. Recently, great strides have been made in this area to produce sensory neurons from human pluripotent stem cells (hPSCs) using combined small molecule inhibition [5,6]. After 10 days these cells begin to express early markers of sensory neurons, including Brn3a and Isl1, similar to the expression profile of non-differentiated MED17.11. These cells are then grown in basic medium supplemented with growth factors and ascorbic acid and subsequently acquire nociceptor transcription factors and functional ion channel expression after a further 2–6 weeks in culture. This is in strong contrast to differentiated MED17.11 cells where we detected nociceptive markers after 1–7 days. Notably, the acquisition of the important nociceptor transcription factor, Runx1 was not detected in hPSCs-derived neurons until 4 weeks of culture in growth factors. In addition, veratridine and ATP sensitivity was not observed until 2 weeks post-differentiation in response to growth factors. Capsaicin responses were only seen after 6 weeks and in just 1–2% of cells. This is in contrast to MED17.11 cells which respond to both ATP (50%) and capsaicin (71%) after just a few days of differentiation. Indeed when we cultured MED17.11 in just NGF and GDNF, we observed little functional differentiation (Fig 4D) compared with using our primary differentiation medium (Fig 4B). Therefore it will be interesting to determine whether our differentiation medium can be applied to neuralised hPSCs to accelerate acquisition of sensory neuron markers. The time saved (days instead of weeks) will have a significant impact on the suitability of MED17.11 or hPSCs derived neurons for high-throughput drug screens. Moreover, immortalising differentiated hPSCs at the same developmental stage as MED cell lines might further simplify the derivation of sensory neurons from hPSCs by eliminating the need to maintain and manipulate stem cells. Recently, Wainger *et al* derived nociceptors from fibroblasts by transfecting them with five transcription factors. Moreover, the fibroblasts were derived from patients with familial dysautonomia [35], thus providing a novel way to model human neuronal disease *in vitro*. This is an extremely encouraging development for the field. But for many basic researchers, a simpler, cheaper and quicker method for deriving sensory neurones in culture is necessary and thus MED17.11 cells may be more suitable for this work.

In summary, MED17.11 cells have the capability of differentiating into sensory neurons of multiple modalities, with particularly strong evidence that they differentiate efficiently into nociceptive-lineage neurons. Moreover, like primary DRG neurons they are sensitive to inflammatory mediators. The advantage of creating a temperature-dependent conditional cell line means that there is a continuous supply of material, but that any confounding effects that the immortalising gene on phenotype can be reduced. MED17.11 cells can be induced to undergo rapid and efficient maturation into sensory neurons using our differentiation medium; enabling large scale preparations for high-throughput and “omic” screens. The use of MED17.11 should aid basic and pharmaceutical research by providing an *in vitro* model to study the molecular mechanisms underlying nociception, neuronal development and phenotype specification, while at the same time reducing the number of animals used to derive primary cultures.

## References

[1] Dray A (1995) Inflammatory mediators of pain. Br J Anaesth 75: 125–131. 7577246 10.1093/bja/75.2.125

[2] Platika D, Boulos MH, Baizer L, Fishman MC (1985) Neuronal traits of clonal cell lines derived by fusion of dorsal root ganglia neurons with neuroblastoma cells. Proc Natl Acad Sci U S A 82: 3499–3503. 3858835 10.1073/pnas.82.10.3499 PMC397804

[3] Wood JN, Bevan SJ, Coote PR, Dunn PM, Harmar A, et al (1990) Novel Cell Lines Display Properties of Nociceptive Sensory Neurons. Proceedings of the Royal Society of London Series B: Biological Sciences 241: 187–194. 1979443 10.1098/rspb.1990.0084

[4] Chen W, Mi R, Haughey N, Oz M, Hoke A (2007) Immortalization and characterization of a nociceptive dorsal root ganglion sensory neuronal line. J Peripher Nerv Syst 12: 121–130. 17565537 10.1111/j.1529-8027.2007.00131.x PMC3417147

[5] Young GT, Gutteridge A, Fox HDE, Wilbrey AL, Cao L, et al (2014) Characterizing Human Stem Cell-derived Sensory Neurons at the Single-cell Level Reveals Their Ion Channel Expression and Utility in Pain Research. Mol Ther 22: 1530–1543. 10.1038/mt.2014.86 24832007 PMC4435594

[6] Chambers SM, Qi Y, Mica Y, Lee G, Zhang XJ, et al (2012) Combined small-molecule inhibition accelerates developmental timing and converts human pluripotent stem cells into nociceptors. Nat Biotechnol 30: 715–720. 10.1038/nbt.2249 22750882 PMC3516136

[7] Jat PS, Noble MD, Ataliotis P, Tanaka Y, Yannoutsos N, et al (1991) Direct derivation of conditionally immortal cell lines from an H-2Kb-tsA58 transgenic mouse. Proceedings of the National Academy of Sciences 88: 5096–5100. 1711218 10.1073/pnas.88.12.5096 PMC51818

[8] Baker MD, Bostock H (1997) Low-threshold, persistent sodium current in rat large dorsal root ganglion neurons in culture. J Neurophysiol 77: 1503–1513. 9084615 10.1152/jn.1997.77.3.1503

[9] Hjerling-Leffler J, AlQatari M, Ernfors P, Koltzenburg M (2007) Emergence of Functional Sensory Subtypes as Defined by Transient Receptor Potential Channel Expression. The Journal of Neuroscience 27: 2435–2443. 17344381 10.1523/JNEUROSCI.5614-06.2007 PMC6672507

[10] Sun Y, Dykes IM, Liang X, Eng SR, Evans SM, et al (2008) A central role for Islet1 in sensory neuron development linking sensory and spinal gene regulatory programs. Nat Neurosci 11: 1283–1293. 10.1038/nn.2209 18849985 PMC2605652

[11] Virginie Neirinckx CcC, Bernard Rogister,Sabine Wislet- Gendebien (2013) Neural Fate of Mesenchymal Stem Cells and Neural Crest Stem Cells: Which Ways to Get Neurons for Cell Therapy Purpose? Trends in Cell Signaling Pathways in Neuronal Fate Decision.

[12] Fournier AE, Takizawa BT, Strittmatter SM (2003) Rho kinase inhibition enhances axonal regeneration in the injured CNS. J Neurosci 23: 1416–1423. 12598630 10.1523/JNEUROSCI.23-04-01416.2003 PMC6742251

[13] Neirinckx V, Coste C, Rogister B, Wislet-Gendebien S (2013) Concise review: adult mesenchymal stem cells, adult neural crest stem cells, and therapy of neurological pathologies: a state of play. Stem Cells Transl Med 2: 284–296. 10.5966/sctm.2012-0147 23486833 PMC3659839

[14] Matsuda S, Uehara Y (1984) Prenatal development of the rat dorsal root ganglia. Cell and Tissue Research 235: 13–18. 6697377 10.1007/BF00213717

[15] Montelius A, Marmigère F, Baudet C, Aquino JB, Enerbäck S, et al (2007) Emergence of the sensory nervous system as defined by Foxs1 expression. Differentiation 75: 404–417. 17309606 10.1111/j.1432-0436.2006.00154.x

[16] Ravenall SJ, Gavazzi I, Wood JN, Akopian AN (2002) A peripheral nervous system actin-binding protein regulates neurite outgrowth. European Journal of Neuroscience 15: 281–290. 11849295 10.1046/j.0953-816x.2001.01862.x

[17] Minett MS, Nassar MA, Clark AK, Passmore G, Dickenson AH, et al (2012) Distinct Nav1.7-dependent pain sensations require different sets of sensory and sympathetic neurons. Nat Commun 3: 791 10.1038/ncomms1795 22531176 PMC3337979

[18] Kim J, Lo L, Dormand E, Anderson DJ (2003) SOX10 Maintains Multipotency and Inhibits Neuronal Differentiation of Neural Crest Stem Cells. Neuron 38: 17–31. 12691661 10.1016/s0896-6273(03)00163-6

[19] Chen C-L, Broom DC, Liu Y, de Nooij JC, Li Z, et al (2006) Runx1 Determines Nociceptive Sensory Neuron Phenotype and Is Required for Thermal and Neuropathic Pain. Neuron 49: 365–377. 16446141 10.1016/j.neuron.2005.10.036

[20] Shibasaki K, Murayama N, Ono K, Ishizaki Y, Tominaga M (2010) TRPV2 Enhances Axon Outgrowth through Its Activation by Membrane Stretch in Developing Sensory and Motor Neurons. The Journal of Neuroscience 30: 4601–4612. 10.1523/JNEUROSCI.5830-09.2010 20357111 PMC6632311

[21] Coste B, Mathur J, Schmidt M, Earley TJ, Ranade S, et al (2010) Piezo1 and Piezo2 are essential components of distinct mechanically activated cation channels. Science 330: 55–60. 10.1126/science.1193270 20813920 PMC3062430

[22] Cox JJ, Reimann F, Nicholas AK, Thornton G, Roberts E, et al (2006) An SCN9A channelopathy causes congenital inability to experience pain. Nature 444: 894–898. 17167479 10.1038/nature05413 PMC7212082

[23] Nassar MA, Stirling LC, Forlani G, Baker MD, Matthews EA, et al (2004) Nociceptor-specific gene deletion reveals a major role for Nav1.7 (PN1) in acute and inflammatory pain. Proc Natl Acad Sci U S A 101: 12706–12711. 15314237 10.1073/pnas.0404915101 PMC515119

[24] Fang X, Djouhri L, McMullan S, Berry C, Waxman SG, et al (2006) Intense isolectin-B4 binding in rat dorsal root ganglion neurons distinguishes C-fiber nociceptors with broad action potentials and high Nav1.9 expression. J Neurosci 26: 7281–7292. 16822986 10.1523/JNEUROSCI.1072-06.2006 PMC6673936

[25] Fjell J, Cummins TR, Dib-Hajj SD, Fried K, Black JA, et al (1999) Differential role of GDNF and NGF in the maintenance of two TTX-resistant sodium channels in adult DRG neurons. Brain Res Mol Brain Res 67: 267–282. 10216225 10.1016/s0169-328x(99)00070-4

[26] Benn SC, Costigan M, Tate S, Fitzgerald M, Woolf CJ (2001) Developmental expression of the TTX-resistant voltage-gated sodium channels Nav1.8 (SNS) and Nav1.9 (SNS2) in primary sensory neurons. J Neurosci 21: 6077–6085. 11487631 10.1523/JNEUROSCI.21-16-06077.2001 PMC6763192

[27] Ueno S, Tsuda M, Iwanaga T, Inoue K (1999) Cell type-specific ATP-activated responses in rat dorsal root ganglion neurons. Br J Pharmacol 126: 429–436. 10077235 10.1038/sj.bjp.0702319 PMC1565824

[28] Teichert RW, Raghuraman S, Memon T, Cox JL, Foulkes T, et al (2012) Characterization of two neuronal subclasses through constellation pharmacology. Proc Natl Acad Sci U S A 109: 12758–12763. 10.1073/pnas.1209759109 22778416 PMC3411979

[29] Barnes S, Hille B (1988) Veratridine modifies open sodium channels. J Gen Physiol 91: 421–443. 2454286 10.1085/jgp.91.3.421 PMC2216135

[30] Kobayashi K, Fukuoka T, Obata K, Yamanaka H, Dai Y, et al (2005) Distinct expression of TRPM8, TRPA1, and TRPV1 mRNAs in rat primary afferent neurons with adelta/c-fibers and colocalization with trk receptors. J Comp Neurol 493: 596–606. 16304633 10.1002/cne.20794

[31] Marmigere F, Ernfors P (2007) Specification and connectivity of neuronal subtypes in the sensory lineage. Nat Rev Neurosci 8: 114–127. 17237804 10.1038/nrn2057

[32] Molliver DC, Snider WD (1997) Nerve growth factor receptor trkA is down-regulated during postnatal development by a subset of dorsal root ganglion neurons. The Journal of Comparative Neurology 381: 428–438. 9136800 10.1002/(sici)1096-9861(19970519)381:4<428::aid-cne3>3.0.co;2-4

[33] Vetter I, Lewis RJ (2010) Characterization of endogenous calcium responses in neuronal cell lines. Biochemical Pharmacology 79: 908–920. 10.1016/j.bcp.2009.10.020 19883631

[34] Kusano K, Gainer H (1993) Modulation of voltage-activated Ca currents by pain-inducing agents in a dorsal root ganglion neuronal line, F-11. Journal of Neuroscience Research 34: 158–169. 8383774 10.1002/jnr.490340203

[35] Wainger BJ, Buttermore ED, Oliveira JT, Mellin C, Lee S, et al (2015) Modeling pain in vitro using nociceptor neurons reprogrammed from fibroblasts. Nat Neurosci 18: 17–24. 10.1038/nn.3886 25420066 PMC4429606

